# Multi-(myco)toxins in Malting and Brewing By-Products

**DOI:** 10.3390/toxins11010030

**Published:** 2019-01-09

**Authors:** Kristina Mastanjević, Jasmina Lukinac, Marko Jukić, Bojan Šarkanj, Vinko Krstanović, Krešimir Mastanjević

**Affiliations:** 1 Faculty of Food Technology Osijek, Josip Juraj Strossmayer University of Osijek, F. Kuhača 20, 31000 Osijek, Croatia; ptfosptfos2@gmail.com (J.L.); ptfosptfos@gmail.com (M.J.); vkrstano@ptfos.hr (V.K.); kmastanj@gmail.com (K.M.); 2 Department of Food Technology, University North, University Center Koprivnica, Trg dr. Žarka Dolinara 1, 48000 Koprivnica, Croatia; bsarkanj@unin.hr

**Keywords:** Multi-toxins, mycotoxins, malting and brewing by-products, animal feed

## Abstract

Fungi, yeasts, and bacteria are common microorganisms on cereals used in malting and brewing industries. These microorganisms are mostly associated with the safety and quality of malt and beer, but also with the health safety of by-products used in animal nutrition. The real problem is their harmful metabolites—toxins that, due to their thermostable properties, can easily be transferred to malting and brewing by-products. Besides fungal metabolites, other toxins originating from plants can be harmful to animal health. Precise and accurate analytical techniques broadened the spectrum of known toxins originating from microorganisms and plants that can pose a threat to animal health. Multi-(myco)toxin analyses are advanced and useful tools for the assessment of product safety, and legislation should follow up and make some important changes to regulate yet unregulated, but highly occurring, microbial and plant toxins in malting and brewing by-products used for animal feed.

## 1. Introduction

Beer can be produced not only from barley malt, but other cereals (malted or unmalted wheat, corn, rice, sorghum) can be added to the brew to cut production costs or improve the colloidal stability of beer. During malting and brewing processes, by-products are formed and adjusted to be reused as feed. Nowadays, modern processes can be applied and transform these by-products into functional foods or additives for the food industry. Many studies on myco- and other toxins are available in professional and scientific literature, but only a few investigations have focused on by-products formed during the malting and brewing process, such as germ and rootlets, spent grains, and spent yeast. These by-products represent a nutritious and valuable, low-cost source of feed for livestock. In terms of using it as functional food, malting and brewing (M/B) by-products can be added to different cereal products to enhance their nutritional properties. However, the above-mentioned by-products can also be contaminated with mycotoxins and other toxins originating from plants or other microorganisms. Most mycotoxins are thermo-stable and can survive the unit operations applied during the malting and brewing processes. In addition, M/B by-products are susceptible to microbial contamination and have a short shelf life (due to the original high water content). To ensure the food safety for the final consumers, whether animals or humans, it is necessary to investigate, determine, and regularly monitor the existing toxins in these by-products.

Encouraged by the recent findings of our research on multi-toxins in malting and brewing by-products [1,2], we decided to round up similar papers and point out how important and urgent this matter of emerging myco- and other toxins in animal feed is.

## 2. The Production of Malting and Brewing By-Products

Malting is a process of forced germination of cereal grains to develop enzymes needed to break down starch and proteins to more simple molecules (glucose, maltose, maltotriose, maltodextrines, and aminoacids), so that yeast can be employed in alcohol production. When germ and rootlets are shown, germination is halted by hot air to dry out the excess water. After that, the germinated grain is subjected to degermination, germ and rootlets get separated, and are adapted to be sold as animal feed. Since they are low in bulk density, they are mixed with barley and malt dust and small barley grains and pelletized [3]. Fungi originating from field and storage facilities thrive under malting conditions.

The brewing process consists of several continuous phases. Firstly, milled malt is subjected to mashing (slow heating with water). The residual precipitate from this process is called spent grains and the liquid part, wort, undergoes further processing to become beer.

After precipitation, wort is boiled, and, at this point, hops are added. When boiling is done, hopped wort is cooled and separated from hop residues/spent hops. Spent hops get discarded, but some of it ends up in the trub/cold break (the protein precipitate leftover after cooling of wort).

After cooling, hopped wort is inoculated with yeast and left for main fermentation in a fermentation vessel. During this time, the yeast biomass increases three- to six fold, and at the end of this phase, the yeast biomass is called spent yeast [4].

All these by-products can be utilized for human or animal consumption. However, the native microflora and accompanying produced or bio-transformed (myco)toxins can be a cause of serious economy- and health-related problems for humans and animals.

## 3. Common Fungal Species in Malting and Brewing

**Figure 1:**
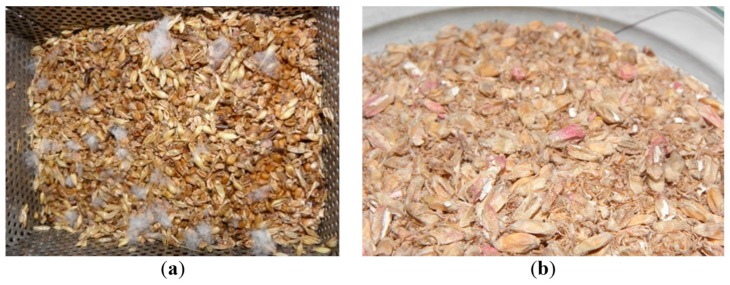
Heavily infected malt (*F. graminearum*) before (**a**) and after (**b**) kilning.

The most common fungal species in malting and brewing by-products are different Fusarium species. *F. acuminatum*, *F. anthophilium*, *F. avanceum*, *F. cerealis* (crookwellense), *F. chlamydosporum*, *F. culmorum*, *F. equiseti*, *F. graminearum*, *F. heterosporum*, *F. nygamai*, *F. oxysporum*, *F. poae*, *F. proliferatum*, *F. sambucinum*, *F. semitectum*, *F. sporotrichoides*, *F. subglutaminans*, *F. tricintum*, and *F. verticilioides* all belong to genus *Fusarium*, but only some of them show inclination to invade grains in our region. According to Krstanović et al. [5], the most prevalent species in Eastern Croatia is *F. graminearum*, being present in both wheat and barley samples (Figure 1). The highest contaminations (20%) were detected in samples from Nova Gradiška. *F. culmorum* was found in significantly lower percentages, with 3.1% being the highest contamination, and statistically did not represent any threat to human health. Generally, colder Northern European regions support the growth of *F. culmorum*, and warmer parts [6] of Europe fight battles with *F. graminearum*. However, the global climate changes caused a shift in Fusarium species’ distribution. Namely, according to Parikka et al. [7], *F. culmorum* lost its place in being the prevalent specie in Nordic countries. *F. graminearum* is expected to populate the Northern European areas very soon. Ward et al. [8] reported on toxicity of *F. graminearum* in North America and similar research was conducted in China, where the results indicated that more aggressive isolates (producing a wider variety of mycotoxins) are taking over [9].

Other fungal species invading cereals and making problems in malting and brewing industries include: *Alternaria* spp., *Epicoccum* spp., *Penicillium* spp., *Aspergillus* spp., etc. In research conducted on Slovakian barley, many fungal species were detected: *Acremonium strictum*, *Alternaria* spp., *Aspergillus niger*, *Aspergillus flavus*, *Botrytis cinerea*, *Cladosporium herbarum*, *Cladosporium cladosporioides*, *Cochliobolus sativus* (*Helminthosporium sativum*), *Epicoccum nigrum*, *Fusarium* spp., *Chrysonilia sitophila*, *Microdochium nivale*, *Mucor* sp., *Nigrospora sphaerica*, *Penicillium* sp., *Phaeosphaeria nodorum* (*Septoria nodorum*), *Pyrenophora teres* (*Helminthosporium teres*), *Rhizoctonia* sp., *Rhizopus nigricans*, *Stemphylium* sp., *Tanatephorus cucumeris* (*Rhizoctonia solani*), *Trichoderma* sp., and *Verticillium albo-atrum* [10].

In a study conducted by Gonzalez Pereyra et al. [11], the toxigenic species belonging to the genera, *Fusarium*, *Aspergillus*, *Penicillium*, *Cladosporium*, *Geotrichum,* and *Alternaria*, were identified in malted barley and brewer’s spent grains. The detected *Fusarium* spp. made 30% and *Aspergillus* spp. made 27.3% of contamination, and 30% of brewer’s grain samples were contaminated with *Mucorales*. Most prevalent *Fusarium* species in brewer’s spent grain were *Fusarium verticillioides* (50%) and *Fusarium proliferatum* (25%) while *Fusarium equiseti* (12.5%) and *Fusarium oxysporum* (12.5%).

Piacentini et al. [12] published the results from their research involving the detection of fungal species and mycotoxins (deoxynivalenol (DON) and fumonisins (FB_1_ and FB_2_)) on malting barley. They also reported that the most prevalent genera were *Fusarium*. *F. graminearum* and *F. verticillioides* were the dominant species, with an incidence of occurrence of 26% and 12%. Other fungal genera determined in their research included *Penicilium*, *Alternaria*, and *Rhizophus*. Mycotoxin analysis revealed that DON contaminated 18% of samples while FB_1_ and FB_2_ were detected at a lesser extent, 10% for FB_1_ and 1% for FB_2_.

Fungi on malting barley are problematic because of the favourable conditions [13], which support fungal mycelium growth and mycotoxins’ production. Fungi-thriving process conditions are assisted by the easily available nutrients from the grain, emergence in water, exposure to lower temperatures (10–14 °C) and aeration, high air humidity (>90%), and overall high grain moisture during germination and the first phase of kilning (cca. 45% grain moisture). Subsequent mycotoxin biosynthesis occurs at almost every stage of malting and brewing. Colder temperatures during steeping and germination enable fungi to produce mycotoxins. Mycotoxins synthesis occurs [2,14] during the whole malting process, especially after kilning, and continues during brewing. The fermenting stage also allows mycotoxins’ content increase. In case there are some leftover spores, mycotoxins can be found even during aging. Some toxins stem from yeast (tryptophol) and are produced after the inoculation of wort. According to our findings [2], mycotoxin concentrations vary during the brewing stage, and after inoculation, mycotoxins (mainly DON) can show a significant increase in comparison to wort.

## 4. General Toxicity and Occurrence of Multi- and Myco-Toxins

Fungal infections are not unusual and they often contaminate cereals, diminishing their quality and lowering their marketability. However, the most important problem related to fungi is the occurrence of different myco- and other toxins that, directly or indirectly, affect human and animal health.

The sources of human exposure to mycotoxins can be direct (from plant-derived foods contaminated with toxins) or indirect (through a carryover of mycotoxins and their metabolites in animal products, such as meat and eggs) [15]. The exposure to air and dust containing toxins can also cause serious consequences to human health [16]. Therefore, it is important to wear suitable protection (masks-preferably with HEPA (High Efficiency Particulate Air) filter, goggles) during handling of infected grain [17].

As is well-known, mycotoxins influence vital organs and tissues, having carcinogenic properties, inducing immunosuppression and reproductive problems in animals and humans [15]. Most mycotoxins affect the health of humans and animals by causing nephropathy, infertility, cancer, or death [18]. Intense research on mycotoxins reveals that cca. 400 mycotoxins are known till today [19] and about 200 of them belong to the group of trichothecenes [18]. Especially dangerous are mycotoxins that accumulate in animal tissues, meat, and fat, because they can cause problems when ingested by humans.

Some studies report aflatoxins, fumonisins, trichothecenes, ochratoxin A, and zearalenone as the most significant mycotoxin in the barley-beer chain [20,21], but for animal feed, only seven mycotoxins: Aflatoxin B_1_ (AFB_1_), DON, zearalenone (ZEA), fumonisins (FUM) and ochratoxin A (OTA), T-2, and HT-2 are regulated by the EU (European Union) legislation [22,23,24] and are under strict governmental control.

Aflatoxins (AFs) are produced by different species of *Aspergillus* fungi (*A. flavus*, *A. parasitous*, and *A. nomius*). They can occur in different foods (groundnuts, tree nuts, maize, corn, rice, figs, and other dried foods, spices, crude vegetable oils, and cocoa beans) as a result of fungal contamination before and after harvest. Aflatoxins are potentially toxic, carcinogenic, mutagenic, and immunosuppressive compounds [25]. There are 18 different types of AFs, with the major members being AFB_1_, AFB_2_, AFG_1_, AFG_2_, AFM_1_, and AFM_2_. AFB_1_ is the only mycotoxin whose maximum content in mg/kg (relative to a feedstuff moisture content of 12%) is determined by the Commission Directive 2003/100/EC [22]. Even though AF’s can end up in beer, the European Union has not set a maximum allowable limit for AFB_1_ in beer, although there is a limit for barley and malt [26]. AFB_1_ has been described as a high carcinogenic natural toxin and has been classified as a Group 1 (carcinogenic to humans) [27]. AFB_1_ can be found in beer [28,29,30] and raw materials, such as barley [31,32] and corn [33,34], used for the production of beer. Thus, AFB_1_ can be found in brewing by-products that can be used as animal feed and cause health problems for animals, as confirmed by Gerbaldo et al. [35] and Gonzalez Pereyra et al. [11].

Most *Fusarium* spp. produce trichothecenes, and amongst them, *F. graminearum*, *F. culmorum*, *F. proliferatum*, and *F. equiseti* are characterized as main producers of trichotecenes [36]. However, many other fungal species can produce trichotecenes: *Myrotecium*, *Trichoderma*, *Cephalosporium*, *Verticimonosporium*, and *Stachybotrys* [37]. According to Krstanović et al. [5], the most prevalent species on cereals in Eastern Croatia are *F. graminearum* Schw. (teleomorph *Gibberella zeae*) and *F. culmorum* (Wm. G. Sm.) Sacc. (teleomorph unknown) can be found at a lesser extent. DON, being the most prevalent mycotoxin in cereals, is identified as one of the indicators of the quality and safety of barley and wheat used for malting [38]. In the research by Velić et al. [39] conducted with *F. graminearum*, a correlation between fungal contamination and DON appearance was shown. DON is identified as a protein synthesis inhibitor in eukaryotic cells due to its ability to bind to ribosomes. Low to moderate concentrations of DON can affect the health of animals, causing anorexia (lower economical profit), and higher doses can cause vomiting [40,41]. Besides DON, other trichotecenes can be found in malting and brewing by-products, and many of them occur in coherence with their derives. Many trichotecenes are well described in scientific literature and are well known in popular circles. They are nivalenol (NIV), T-2 toxin, diacetoxyscirpenol (DAS), etc. However, they have not been yet included into legislative regarding barley, malt, or beer for that matter. This can represent a serious problem since many of them can be found in malting and brewing by-products used as animal feed [1,2].

ZEA is one of the infamous mycotoxins that acts similarly to estrogen and is related with clinical manifestations of various estrogenic effects in humans and farm animals. The exposure to this mycotoxin can been seen in pubertal changes in young children in Puerto Rico [42] and gynecomastia with testicular atrophy in southern Africa [43]. Although ZEA is mostly determined in maize, it can easily be found in other cereals, such as wheat and barley. Maize is one of the unmalted cereals that can be added to a brew to cut costs and improve the colloidal stability of beer, and thus can cause damage to the brewing industry if ZEA is present in the raw material. ZEA has different modified forms that derive from two different metabolic phases:

1. Phase I includes: α-zearalenol, β-zearalenol (α-ZEL and β-ZEL), zearalenone-4-O-β-glucoside (ZEA-14Glc), zearalenone-16-O-β-glucoside (ZEA-16Glc), and zearalenone sulphate (ZEA-14-sulphate, ZEA-14S), and many of them have the ability to transform into their basic form during digestion in the mammalian intestinal tract [44]. ZEA-14Glc can generate complex compounds, such as zearalenone-malonyl-glucoside (ZEA-MalGlc), zearalenone-di-hexoside (ZEA-di-hexoside), and zearalenone-pentosylhexoside [45].
2. Phase II derivate, β-zearalenol-4-glucoside (β-ZEL-4Glc), may be formed by maize itself during xenobiotic detoxification [46] and can easily be transferred back into the original form-zearalenone [47].

Binder et al. [48] conducted research in which they monitored the biotransformation of two plants and one fungal metabolite: ZEN-14-sulfate, ZEN-14-O-β-glucoside, and ZEN-16-O-β-glucoside in pigs. Although the total biological recoveries in urine and faeces were below 50%, the authors attributed this to the extensive metabolization by intestinal bacteria to yet unknown metabolites and offered in conclusion that the complete hydrolyzation of all monitored metabolites (ZEN-14-sulfate, ZEN-14-O-β-glucoside, and ZEN-16-O-β-glucoside) occurred, contributing to the total ZEN toxicity.

Fumonisins are mycotoxins produced by fungi belonging to the genus, *Fusarium*. There are many detected fumonisins, but type B fumonisins are most prevalent in feed and food chains [49]. Fumonisins B_1_, B_2_, B_3_, and B_4_ all comprise this group, with FB_1_ and FB_2_ being the most common members [50,51]. Although primarily found in corn, fumonisins can be found in other cereals. Symptoms of fumonisin intoxication are various [52] for various animals. Pigs, for example, can suffer from porcine pulmonary edema [53] and horses can manifest hemorrhagic-liquefactive brain lesions. In humans, neural tube defects and growth retardation in children can occur [54]. According to Voss and Riley [52], FB_1_ is characterized as a potent carcinogen as some reports link exposure to high doses of FB_1_ to human esophageal cancers [55]. However, no strong empirical relationship has been established between the manifestation of cancer and FB_1_, so FB_1_ is still being considered as possibly carcinogenic to humans and is categorized as a group 2B carcinogen [56]. Fumonisins can also exist in modified forms, which Braun and Wink [56] call “cryptic” fumonisins. They form when basic fumonisins undergo hydrolyzation, usually during digestion in the gastrointestinal tract. In 2018, EFSA (European Food Safety Authority) [57] issued a scientific opinion on fumonisins and their modified and hidden forms in feed. According to EFSA, modified forms are extractable from the matrix and can be formed during biotransformations in fungal, plant, or animal organisms or during thermal and chemical processes to which feed and food can be subjected to. The term ‘hidden forms’ refers to the fraction of fumonisins strongly bonded with non-covalent bonds to the matrix and they are basically non-extractable.

Ochratoxins are secondary metabolites primarily produced by *Penicillium* and *Aspergillus* species. This group involves ochratoxin (OTA), its methyl ester, its ethyl ester also known as ochratoxin C (OTC), 4-hydroxyochratoxin A (4-OH OTA), ochratoxin B (OTB) and its methyl and ethyl esters, and ochratoxin α (OTα) [58]. OTA is nephrotoxic, carcinogenic, teratogenic, genotoxic, and immunotoxic [59]. OTA is one of the highest occurring ochratoxins and is incorporated into legislative recommendations and regulations. As reported by Huff and Doerr [60], OTA and aflatoxin can act synergistically and significantly retard the growth of broiler chickens.

T-2 and HT-2 are *Fusarium* mycotoxins incorporated into EU legislative. Like other mycotoxins, their occurrence is climate dependent and they usually occur together. They are toxic to most animals and humans as they interfere with protein synthesis and haematopoiesis inhibition, lymphoid depletion, and necrotic lesions [61]. Plants and yeasts can modify these mycotoxins into different glucosides: T-2 3-O-glucoside (T-2Glc) and HT-2-O-3-glucoside (HT-2Glc), HT-2-di-glucoside (HT-2di-Glc) [62], di-glucosides (T-2di-Glc, and HT-2di-Glc) [63]. Yeasts of the genus, *Trichomonascus* and *Blastobotrandy*, have three biotransformation levels for the T-2 toxin: Acetylation to 3-acetyl-T-2; glycosylation; and the removal of an isovaleryl group from the molecule, leading to the formation of neosolaniol [64]. Other modified forms of T-2 and HT-2 mycotoxins include: HT-2-malonyl-glucoside, hydroxy-HT-2-glucoside, dehydro-HT-2-glucoside, T2-triol-glucoside, and T-2-feruloyl-T-2 toxin [65].

Emerging mycotoxins in food and feed, such as enniatins (ENNs) and beauvericin (BEA), have been under the scientific light for the past few years. The toxicity of enniantin B (ENNB) and BEA has been recently described by a group of authors in EFSA’s [57] external scientific report, where it was reported that ENNB is genotoxic to mice and BEA targeted thyroid, kidneys, and reproductive systems in both sexes. Repeated oral exposure to BEA seems to affect male mice more, but further studies should be conducted.

Besides these mycotoxins and their derivates stemming from plants itself, there are many others that can be found in malting and brewing by-products. Some of them are reported in a recent publication by Mastanjević et al. [1]. Emerging toxins are currently the focal point of the scientific community since these toxins are not included into legislation and some of them are very poorly studied; in a sense, there is little or no information of how they affect human or animal health.

## 5. Multi- and Myco-Toxins in Malting and Brewing By-Products

Since M/B by-products are rich in proteins, digestible fibre material, vitamins, and minerals, their utilization as feed supplements has become an alternative for animal feed [11,66,67]. These by-products represent low cost and nutritious animal feed, but also promote rumen fermentation and digestion and overall act beneficially on animal health [11].

Cavaglieri et al. [67] confirmed the correlation between fungal contamination and natural incidence of selected mycotoxins in barley rootlets. Namely, Cavaglieri et al. [67] found FB_1_ contamination in 100% of barley rootlet samples. However, FB_1_ levels found in this substrate were subjectively high (254–2043 µg/kg); while Gerbaldo et al. [35] carried out another study that confirmed AFB_1_ contamination on brewer’s grain.

In a research conducted by Gonzalez Pereyra et al. [11], the detected mycotoxins in malting barley and BSG (brewer’s spent grain) were fumonisin B_1_ and AFB_1_. Although FB_1_ toxin levels were below the recommended values (104–145 µg/kg), the occurrence of the fumonisins’ contamination in these samples was 100%. Aflatoxin B_1_ was detected in 18% of brewer’s spent grain samples (19–45 µg/kg) and was presumably produced during storage [68]. Other mycotoxins (AFB_2_, AFG_1_, AFG_2_, or ZEA) were not found in the analysed samples. This indicates that, in the case of brewer’s grain and other barley by-products intended for animals, mycotoxin contamination increases with the storage time if storage conditions are not closely controlled. Similar research was conducted by Habschied et al. [69], in which the ZEA concentration in barley germ and rootlets after degermination increased with prolonged storage time and higher water activity (a_w_).

**Figure 2:**
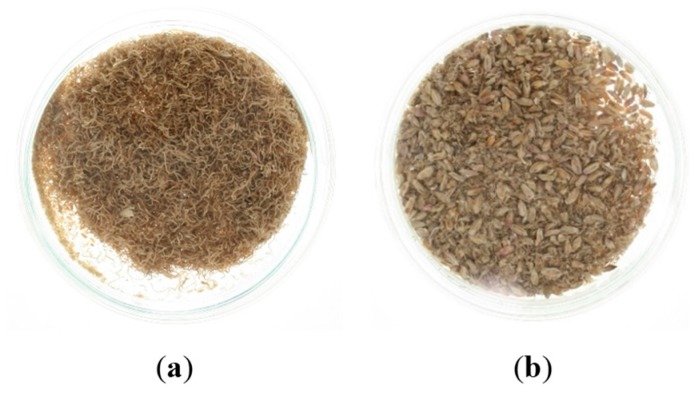
Germ/rootlets of (**a**) healthy malt (**b**) severely infected malt with *F. graminearum*.

Barley germ and rootlets are the result of the ending phase of the malting process [67]. After the final phase of drying, degermination is carried out. Germ and rootlets have a lower water content, but because of their high nutritive value in amino acids and fat content, they are susceptible to microbial degradation. Inappropriate storage and management of these materials can lead to the loss of nutritive substances and mycotoxins concentration increase [67,69]. Figure 2 shows a significant reduction of germ/rootlets in malt severely contaminated with *Fusarium graminearum* (sample b). The higher the initial *Fusarium* contamination, the poorer the germ development will be.

As mentioned before, Mastanjević et al., Habschied et al., and Krstanović et al. investigated several cases in which mycotoxins and multi-toxins were determined in M/B by-products [1,2,69,70,71]. They reported that not only mycotoxins can be found in such by-products, but also in many other toxins and originating from plants or other sources.

**Figure 3:**
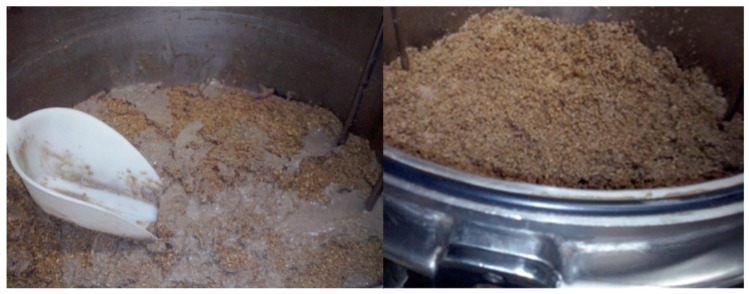
Spent grains and cold break (protein residue) after filtration.

Brewer’s spent grain is obtained after the filtration of mash and are a moist brewing by-product. Spent grain can be used as animal feed in various forms: fresh (wet) (Figure 3), ensiled, or dehydrated (dried) grain [72]. Spent grain is often used as a supplement for lactating cows [73]. Since spent grains and spent yeast are very nutritious, and contain high amounts of available water [11], they are susceptible to microbial growth and are very perishable. Before storage, any excess water must be removed to ensure microbial safety and to prolong the shelf life. Spent yeast also must be treated in additional processes to inactivate the living yeast cells because ruminants have a complex mixture of micro flora to help breakdown the cellulose into simpler carbohydrates they can use for energy. Although small amounts of live yeast can be safe for ruminants, excessive amounts of live yeast cells can disrupt the gut microbiology. This ends up as a gas build up that can block their airways, causing suffocation and even death. Drying also improves the storability of these by-products and enables easier transportation and handling [73].

A research conducted by Mastanjević et al. [2] followed the transition of eight *Fusarium culmorum* mycotoxins (fusarenone-X (FUS-X), 3-ADON, DAS, HT-2, T-2, DON, NIV, ZEA) from starting wheat to beer and associated by-products (steeping water, germ/rootlets, spent grains, and spent yeast). The results showed that steeping water can withhold extremely high DON concentrations amounting up to 20,326 µg/kg. Germ/rootlets also retain high amounts of DON (5636 µg/kg) and NIV (1691 µg/kg). This is in accordance with an earlier research by Krstanović et al. [71] conducted on wheat samples with different initial *F. culmorum* infection levels (0–50%). Namely, germ/rootlets here too retained significantly high DON concentrations (5000–12,600 µg/kg), depending on the infection level. The germ is rich in fats and made of soft tissue, hence it is a place of the most intense fungal growth, which explains why DON levels in germ are so high. Similar research was conducted by Habschied et al. [69] on barley malt where ZEA was the followed mycotoxin. This research showed that ZEA levels in malt bran and germ samples were significantly higher than in malt flour (endosperm) in almost all growing conditions.

This affirms that it is necessary to monitor and determine the exact types and quantity of mycotoxins in cereals, final products, and by-products to ensure the food and feed safety [70]. In addition, according to Habschied et al. [69], storage conditions for these by-products are of immense importance. Thus, attention should be paid to ensure the suitable storage conditions.

Except mycotoxins, many malting and brewing by-products contain other toxins stemming from the plant itself, residual fungicides, or herbicides applied in the field. Namely, plants possess two detoxifying mechanisms (chemical modification and compartmentalization) that help them transfigure (myco)toxins to a less toxic form [47]. To explain this, Berthiller et al. [47] and Rychlik et al. [74] described deoxynivalenol-3-glucoside (D-3-G) as a product of a detoxifying plant mechanism, where glycosylation of DON occurs via a conjugation reaction and the result is D3G. Modified mycotoxins usually co-occur with free mycotoxins [47], and can even exceed the concentration of free form in processed foods and feed [75,76].

**Figure 4:**
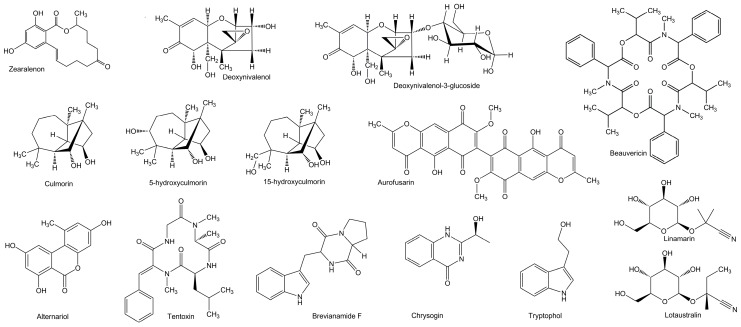
Chemical structure of some of known and emerging toxins.

In research by Mastanjević et al. [1], a wide array of multi-toxins was detected in malting and brewing by-products. Most abundantly found mycotoxins in spent grains and spent yeast were lotaustralin and tryptophol. Other detected myco- and multi-toxins were: Aurofusarin, beauvericin, brevianamid F, chrysogin, culmorin, 5-hydroxyculmorin, 15-hydroxyculmorin, deoxynivalenol, deoxynivalenol-3-glucoside, linamarin, tentoxin, and zearalenon. Some of these toxins can be found in Table 1 and Figure 4.

Since the spectrum of found mycotoxins is very broad, an update regarding the detection and determination of emerging multitoxins in legislation should be made to ensure the health of animals and, subsequently, humans. Surveillance of mycotoxin concentrations should be regular and stricter and analysis spectrum should be expanded, especially on masked mycotoxins (3-acetyl-DON (3-ADON) and 15-acetyl-DON (15-ADON), cyanogenic glucosides (D-3-G), and yeast secondary metabolites (tryptophol).

## 6. Malting and Brewing By-Products Adding Value to Different Food Products

Besides the use in animal feed, malting and brewing by-products can be used to enrich and improve the functional properties of different food products [4,78,79]. Considering the increasing, almost pandemic, occurrence of obesity, diabetes, cancer, and cardiovascular diseases in today’s world, a general idea of functional food derived from food industry by-products has been diligently implemented in contemporary research investigations.

Being produced in large quantities and available all year long, brewing by-products, such as spent grains, spent hops, and spent yeast, can be utilized in food production by adding nutritive value to different products. The main constituents -minerals, nitrogen, and carbon—are the most important compounds from a biotechnological point of view and they have a tremendous potential for use in biotechnological processes. As mentioned before, being rich in carbon and nitrogen makes them suitable for the extraction of sugars, proteins, acids, and antioxidants [4].

Brewer’s spent grain and spent yeast can also be applied in the baking industry as a source of dietary fibers (β-glucans). BSG contains around 17% of cellulose and 28% of non-cellulose polysaccharides (arabinoxylans and lignine). According to some research, BSG retains significant amounts of polyphenols [80,81,82]. Ktenioudaki et al. [83] published an article where brewer’s spent grain can be used as functional food in baked snacks, specifically in breadsticks in this case. However, as reported by the authors, some baking properties were significantly affected and this can be correlated with the high fiber content in BSG. Namely, breadsticks containing BSG were darker, less crispy, and of a lower baking volume. Nevertheless, the quality of breadsticks after 50 days of storage remained unchanged.

From an environmental point of view, the elimination of industrial by-products represents a sane solution to pollution problems and deserves attention.

Much research has been conducted and is still ongoing to reveal and determine the chemistry and biology of mycotoxin production in fungi on many cereals. However, the literature data on mycotoxins in wheat malt and beer are modest and even less data are found on mycotoxins in malting and brewing by-products. Therefore, it is relevant to research and monitor the occurrence of these mycotoxins in wheat food and feed, including malting and brewing products and by-products.

While the FDA proposed a rule on Good Manufacturing Practice, which suggested that separating and selling by-products derived from malting and brewing industries should obligate the manufacturers to honor food safety regulations, the Beer Institute and American Malting Barley Association Inc. strongly disagreed because this would mean additional costs for maltsters and brewers on which they could not agree upon [84].

## 7. The Disposal/Use of Infected Grains

Considering all the risks infected grains can do to the economy in malting and brewing industries, the end question is what to do with infected and contaminated grains. This is rarely mentioned in the scientific papers, and is of great concern for growers, farmers, and M/B industries. However, there are some operations that can reduce the damage caused by fungal infection. Namely, diluting the contaminated grains (only for feed) with healthy ones, removing the outer layer of contaminated grain, and applying chemical and physical decontamination can lessen the detrimental consequences of fungal contamination. Alkaline treatments, binders that can reduce the bioavailability of mycotoxins in the intestinal tract, detoxifiers, and enzymes can be beneficial to toxins’ reduction. Biocontrol is also a possible method. However, the question remains: What to do with heavily infected *Fusarium* grains? Handling the infected grains demands appropriate equipment since grain dust can contain DON and other toxins or fungal spores. During storage and handling, such grains should be excluded from healthy ones and one should be careful not to disperse the spores in the local fields [85]. Badly infected grains could be burnt or possibly used in digestion for biogas or bioethanol production. Composting is also an option. Agnew and Kirsch [86] reported the results from their project, where they discovered that composting seemed to be the safest and most efficient way to eliminate DON from the grains. However, it should be taken into an account that other microorganisms from the composting pile probably modified DON into a less toxic form. Namely, modified forms appear to be less toxic, but when degraded in the gut, they reform into their basic toxic form.

In a nutshell, so far, the most acceptable methods for mycotoxins’ decontamination are [87]:

- Clean and blend—with the uncontaminated grain; exclusively for animal feed;
- Dumping—grains with high toxin levels can be disposed in a bush, slough, or in a hole;
- Gravity sorter—grains infected with *Fusarium* have a lower weight and are easy to sort with this technique;
- Burning—the most common method of disposal. This requires an investment in a grain-burning stove, but the produced heat can be used for heating. However, according to Agnew and Kirsch [86], ash can still contain DON and fungal spores and thus represents a danger to human health;
- Composting;
- Anaerobic digestion; and
- Gamma irradiation [88].

## 8. Conclusions

Literature data indicate that malting and brewing by-products withhold significant amounts of various mycotoxins and for that reason, attention should be paid to ensure the health of animal feed. It is necessary to monitor multi-(myco)toxin concentrations and also to expand the analysis spectrum on masked toxins, since masked mycotoxins can be degraded during digestion by lactic acid bacteria in the intestinal track and recover the toxicity of unmasked mycotoxin [47,89,90]. New masked forms of DON (DON sulphates) that can occur in grains naturally have been confirmed recently [89], but no occurrence or metabolism studies were made. However, in the several research papers Mastanjević et al. published [1,2], the problem of mycotoxins in malting and brewing by-products was referred to.

Mycotoxins are retained in germ/rootlets, spent grains, and spent yeast depending on the genotype susceptibility, starting mycotoxin concentrations, and fungicide treatment [91]. Since the spectrum of found mycotoxins is so broad, as confirmed by Mastanjević et al. [1], this calls for attention and updates on legislation to ensure the health of animals and, subsequently, humans.

According to the literature [68,69], suitable storage conditions for these by-products are indisputably important and should be provided (low a_w_ and temperature and shorter storage time).

## Figures and Tables

**Table 1:** Known and emerging detectable toxins that can be found in M/B by-products.

		Mycotoxins	Source
Fungal	*Fusarium* mycotoxins	Deoxynivalenol, Nivalenol, 3-Acetyldeoxynivalenol, 15-Acetyldeoxynivalenol, Zearalenon, HT-2 toxin, T2-toxin, Moniliformin, Chrysogin, Culmorin,, Aurofusarin, Fumonisin B_1_, Fumonisin B_2_, Fumonisin B_3_, Beauvericin, Enniantins (A, A_1_, B, B_1_)	[1,2,12,15,77]
	Other species mycotoxins (*Aspergillus*, *Alternaria*)	Aflatoxin B_1_, Tentoxin, Brevianamid F	[1,11,77]
Yeast metabolite		Tryptophol	[1]
Plant metabolites		DON-3-glucoside, Lotaustralin, Linamarin	[1,77]
